# Chemical Composition and Antibacterial Activity of Essential Oil of *Cosmos bipinnatus* Cav. Leaves from South Africa

**Published:** 2014

**Authors:** Olufunmiso Olajuyigbe, Anofi Ashafa

**Affiliations:** *Department of Plant Sciences, University of the Free State, Qwaqwa Campus Private Bag X13, Phuthaditjhaba 9886 South Africa. *

**Keywords:** Antibacterial, Bacterial infections, *Cosmos bipinnatus*, Monoterpenes, Sesquiterpenes

## Abstract

The chemical composition of essential oils isolated from the leaves of *Cosmos bipinnatus* and its antibacterial activity were analyzed by GC-MS and microbroth dilution assay respectively. The essential oil extracted from this plant was predominantly composed of monoterpenes (69.62%) and sesquiterpenes (22.73%). The antibacterial assay showed that the oil had significant inhibitory effects against both Gram-negative and Gram-positive bacteria isolates. The MIC of Gram-positive strains ranged between 0.16 and 0.31 mg/mL while those of Gram-negative bacteria ranged between 0.31 and 0.63 mg/mL. The Gram-positive bacteria were more susceptible to the essential oil than the Gram-negative bacteria. Most of the major components of this oil in other plants have been reported for antimicrobial activities. The antibacterial activity can be attributed to effects of the combination of several components of the oil. The results indicate that the *C. bipinnatus* might be exploited as natural antibacterial agent and have application in the treatment of several infectious diseases caused by these bacteria. Since this species is endemic to the eastern Free State, the plant could be collected during its bloom and used efficiently in the management of bacterial infections in South Africa.

## Introduction

Essential oils are complex volatile mixtures of 20-80 constituents existing at significantly low concentrations in plants. Their chemical composition depends on climatic, seasonal and geographic conditions, harvest period and distillation technique. The versatile compositions of essential oils, broad spectrum of antimicrobial potentials and low level of toxicity (1) have enhanced their widespread use in perfumes, pharmaceutical and cosmetic industries as well as food preservatives and additives (2). They are capable of inhibiting food borne pathogens and extend the shelf-life of processed food (3-5). 

Essential oils possess a wide range of pharmacological activities. The antibacterial activities of essential oils and their derivatives have been widely documented (6-9). Schilcher (10), Longbottom *et al.* (11) and Sonboli *et al. *(12), also, indicated that they are essential in herbal medicine. In many studies, their antibacterial activity depended on the type, composition, concentration, processing and the storage conditions of essential oils as well as the type and inoculums sizes of the target microorganisms (13-15). While there are several reports on the composition and antibacterial activities of essential oils from plants, there is a dearth of information on the composition and antibacterial activities of *Cosmos bipinnatus*.

*Cosmos bipinnatus* Cav., known as the garden cosmos or Mexican aster, is a genus within the Asteraceae family. It is a medium sized flowering, herbaceous and half-hardy annual medicinal plant which may reappear through self sowing for several years. While the simple leaves are finely cut and thread-like, they are pinnately cut into deep lobes (16). The plant varies from 0.6 to 1.8 m in height with an open and sprawling habit (17). It has showy solitary pink, purple, red or white flowers that are 5-8 cm in diameter and up to 0.1 m in some selections. Flowering is best in full sun, although partial shade is tolerated. Pharmacologically, certain triterpene alcohols such as helianol from other Compositae were reported to possess anti-inflammatory activity (18) while the use of this plant in the treatment of various diseases like jaundice, intermittent fever, splenomegaly and protection against oxidative DNA damage has been reported (17,19,20). In the traditional medicine of the Basotho, the plant is used to manage headache, pain and stomach disorders. The Afrikaans used it against bed bugs and lice indicating its insecticidal property. Apart from ecology and morphological descriptions on this plant species, there is no scientific information to validate its medicinal potentials. The present study, therefore, aimed at investigating the chemical composition and the antibacterial potential of the essential oil of *C. bipinnatus* using GC-MS analysis and 96 wells microplate methods.

## Experimental

*Plant collection and identification*

*C. bipinnatus* Cav. was collected in March, 2012 from a single population growing on a road construction dump site at Harrismith 9880 (S 28^o^ 17.074'; E 29^o^ 07.952') in the eastern Free State, South Africa. The annual rainfall of the areas is about 750 to 1300 mm and temperature range of 17 to 22 ^o^C. The species was authenticated at the department of Plant Sciences, University of the Free State, Qwaqwa Campus. Herbarium voucher specimen (AshMed/01/2012/QwaHb) was prepared and deposited at the University herbarium. 

*Isolation of essential oil and GC-MS analysis*

About 330 g of the fresh leaves of *C. bipinnatus *was subjected to hydrodistillation for 4 h in Clavenger-type apparatus and the oil was collected in *n*-hexane. The oil was analyzed using a Hewlett Packard 6890 Gas Chromatograph linked with Hewlett Packard 5973 mass spectrometer system equipped with a HP5-MS capillary column (30 m x 0.25 mm, film thickness 0.25 μm, Agilent Technologies Wilmington, DE, USA). The oven temperature was programmed from 50 – 250 °C at a rate of 5 °C/min and pressure at 16.0 kPa. The ion source was set at 200°C with ionization voltage of 70 eV and interface temperature of 250 °C. Helium was used as the carrier gas. Spectra were analyzed using the Hewlett-Packard Enhanced Chem Station G1701 programme for windows.

*Identification of compounds*

The components of the oil were identified by comparing their spectra and retention time with those of the Wiley 275 library (Wiley, New York) in the computer library and literature (21-23). Percentage composition was calculated using the summation of the peak areas of the total oil composition.

*Test organisms*

Four (4) Gram-positive bacteria including *Staphylococcus aureus *ATCC 6538,* Staphylococcus aureus* OK_2a_,* Staphylococcus aureus* OK_2b_, *Bacillus pumilis *ATCC 14884 and six (6) Gram-negative bacteria including *Escherichia coli* ATCC 8739, *Shigella flexneri *KZN*, Shigella sonnei *ATCC 29930,* Proteus vulgaris* CSIR 0030, *Enterobacter faecalis* KZN and *Acinetobacter calcaoceuticus anitratus *CSIR were used in this study. These bacterial isolates were obtained from the Department of Biochemistry and Microbiology, University of Fort Hare, South Africa. The organisms were maintained on nutrient agar plates and were revived for bioassay by subculturing in fresh nutrient broth (Biolab, Johannesburg, South Africa) for 24 h before being used.

*Determination of Antibacterial activity using microdilution assay method*

The minimum inhibitory concentration (MIC) values of the essential oil on each organism were determined using microplate dilution method (24), with slight modifications. Briefly, bacterial strains were cultured overnight (24 h) in sterile nutrient broth (Biolab, Johannesburg, South Africa). The inocula of the bacterial strains were prepared from the overnight broth cultures and suspensions were adjusted to 0.5 McFarland standard turbidity of 10^6^ cfu/mL (25). The oil was dissolved in acetone to increase its miscibility before being used to prepare concentrations ranging from 0.08 - 10.00 mg/mL. Aseptically, the adjusted inocula were used to inoculate the 96-well microtitre plates containing the two fold serial dilutions of the oil. The plates were incubated at 37 ºC and examined after 24 h. To indicate bacterial growth, 40 μL of 0.2 mg/mL of p-iodonitrotetrazolium (97% purity, Sigma, South Africa) solution was added to each well and incubated for 30 min at 37 ºC. The colourless tetrazolium salt was reduced to a red-coloured product by the biological activity of the organisms. Each treatment was performed in triplicate and complete suppression of growth at a specific concentration of oil was required for it to be declared active (24). Moxypen, a brand of penicillin, was used as positive control in the experiment with pure solvent and sample free solutions as blank controls.

## Results

*Chemical composition*

The yield of the *Cosmos bipinnatus* leaf essential oil extracted through hydrodistillation using Clevenger apparatus was 1 mL (0.30%) v/w and the results are presented in Table 1. From the GC-MS analysis, 34 compounds representing 97.89% of the total essential oil composition of *C. bipinnatus *were identified. The major components of the essential oil from this plant are monoterpenes (69.62%) and sesquiterpenes (22.73%). The relative amounts of individual components of the essential oil were, however, expressed as percentages of the peak area relative to the total peak area.

**Table 1:** Chemical composition of *Cosmos bipinnatus* leaf essential oil

**S/no**	**Compound **	**R/time (min)**	**% Composition**
1	2-(Cyclohex-2-enylidene)ethyl ethenoate	8.00	0.15
2	2,5-Cyclohexadiene-1-carboxylic acid,	9.75	0.11
3	α.-Thujene	10.56	0.21
4	Sabinene	11.28	9.35
5	β-pinene	11.46	1.08
6	β.-Myrcene	11.76	0.88
7	α.-Terpinene	12.70	0.23
8	α-Phellandrene	12.96	1.67
9	cis-Ocimene	13.27	1.71
10	(E)-β - Ocimene	13.64	50.23
11	γ.-Terpinene	14.04	0.61
12	Terpinolene	14.91	0.10
13	Mentha-1,4,8-triene	15.99	0.55
14	p-Mentha-1,5,8-triene	16.27	2.54
15	2-Propanol	16.53	0.12
16	Terpinene-4-ol	17.89	3.04
17	3-Buten-1-ol	19.17	0.10
18	(-)-α-copaene	23.46	0.10
19	(-)-.β.-Elemene	23.78	0.22
20	trans-Caryophyllene	24.66	0.29
22	Propanedinitrile	25.58	0.10
23	γ.-Cadinene	26.03	0.43
24	Carbamimidothioic acid,	26.14	0.17
25	Germacrene-D	26.23	13.99
26	Aromadendrene	26.46	0.34
27	α.-Farnesene	26.62	3.15
28	1,5,9-Cyclododecatriene	26.70	0.10
29	3-Buten-2-ol	26.99	0.14
30	β.-cadinene	27.08	1.32
31	Furanone	27.19	0.10
32	α.-copaene-8-ol	29.75	0.23
33	α - Cadinol	30.12	4.27
34	Valerophenone	35.53	0.16
	**Total**		**97.89%**
35	Unknowns		1.94

T/time = retention time

*Antibacterial activity of the essential oil of C. bipinnatus*

The results from the antibacterial study of *C. bipinnatus* essential oil against selected bacteria are presented in Table 2. The oil from this plant inhibited all the tested bacteria at minimum inhibitory concentrations (MIC) ranging from 0.16 - 0.63 mg/mL. The MIC of Gram-positive strains ranged between 0.16 and 0.31 mg/mL while those of Gram-negative bacteria ranged between 0.31 and 0.63 mg/mL. The Gram-positive bacteria were more susceptible to the essential oil than the Gram-negative bacteria. *Staphylococcus aureus* (OK_2a_ and OK_2b_) had MIC of 0.16 mg/mL while *S. aureus* ATCC 6538 was inhibited at 0.31 mg/mL. *Escherichia coli* ATCC 8739 and *Shigella sonnei* ATCC 29930 were inhibited at 0.63 mg/mL while other Gram-negative bacteria, *Proteus vulgaris *CSIR 0030, *Enterobacter faecalis* KZN and *Shigella flexineri* KZN, were inhibited at 0.31 mg/mL. Though the MICs range between 0.16 and 0.63 mg/mL for the two groups of bacteria, eight out of the ten isolates had MICs less than 0.5 mg/mL.

**Table 2:** Activity of *Cosmos bipinnatus* leaf essential oil against bacteria of clinical importance

**Tested bacteria isolates **	**MIC**	**Moxypen**
	**(mg/mL)**	
*Escherichia coli* ATCC 8739	0.63	< 0.09
*Proteus vulgaris* CSIR 0030	0.31	< 0.09
*Enterobacter faecalis* KZN	0.31	1.56
*Shigella sonnei* ATCC 29930	0.63	< 0.09
*Shigella flexneri* KZN	0.31	< 0.09
*Acinetobacter calcaoceuticus anitratus* CSIR	0.31	0.39
*Bacillus pumilis* ATCC 14884	0.16	< 0.09
*Staphylococcus aureus* OK_2a _	0.16	< 0.09
*Staphylococcus aureus* ATCC 6538	0.31	< 0.09
*Staphylococcus aureus* OK_2b_	0.16	< 0.09

## Discussions

For centuries, extracts from many plants used as flavouring and seasoning agents in foods and beverages have been used therapeutically (26, 27). The antimicrobial activities of essential oils used as flavouring agents for years (3, 28, 29) have been due to the oil components or sulphur-containing compounds in the aqueous phase (29, 30). In cognisance of the therapeutic potentials inherent in essential oils of plants, their chemical compositions are determined to elucidate the effectiveness of their bioactive constituents in antibacterial activities. Hence, several oil components have been identified and used in determining antimicrobial activities of plant species from which they were extracted. 

In this study, the essential oil of *C. bipinnatus* was basically made up of monoterpenes and sesquiterpenes. Of these components, (E)-β - Ocimene (50.23%), germacrene D (13.99%), sabinene (9.35%), α-cadinol (4.27%), α-farnesene (3.15%) and terpinene-4-ol (3.04%) were considered the most significant. This is contrary to β-elemene (15-17%), β-caryophyllene (15-17%), germacrene D (10-21%) and bicyclogermacrene (12-15%) indicated as the major components of *C. bipinnatus *by Menut *et al.* (31). The differences in the major components of the essential oil of this plant, from the different locations, could be attributed to their geographical locations. However, while the degree of the antibacterial activity of *C. bipinnatus* could be attributed to the presence of these constituents in high percentages in the oil, the antibacterial activities of these components have been indicated in other plants (2, 32). Sutton *et al.* (33) and Rai *et al.* (34) reported the presences of these components in essential oils of other plants. Melliou *et al.* (35) and Delamare and Moschem-Priscorella (36) indicated the importance of α-pinene, β-pinene, p-cymene and 1, 8-cineole in the antibacterial activity of essential oil. Carson *et al.* (37) and Cuaron *et al.* (38) indicated terpine-4-ol as the principal antimicrobial agent in tea tree oil. Hence, there is a relationship between the oil components and the pharmacological activity of *C. bipinnatus*.

Also, the minimum inhibitory concentrations (MICs) results showed that the essential oil of *C. bipinnatus* presented a significant antibacterial activity against both Gram-positive and Gram-negative bacteria. Although the antibacterial activity of the essential oil could have resulted from combinatory effects of the different components (39) and this essential oil was considered a strong inhibitor of bacterial growth since 80% of the MIC values were lower than 500 µg/mL (40), its antibacterial activity could also be attributed to the main constituents such as (E)-β - Ocimene (50.23%) and germacrene D (13.99%). The degree of the antibacterial activities of the essential oil could be attributed to the hydrophobicity of the components (8) allowing the oil to partition the lipids of the bacterial cell membrane, making them more permeable while causing leakages of cellular constituents and ions (41,42). Although the oil components were able to infiltrate the cells and interact with cellular metabolic mechanisms (13), the Gram-negative bacteria were considered less susceptible because they possess cell membrane restricting the diffusion of hydrophobic compounds through its lipopolysaccharide covering (43). While the permeable cell membrane rendered Gram-positive bacteria more susceptible (44), the outer complex membrane in Gram-negative bacteria cell wall prevented interaction of the bacteria cell with harmful oil components (45). 

In conclusion, essential oils can be a source of a great diversity of chemical components equipped with antimicrobial capacity. The GC-MS analysis of the oil extract from *C. bipinnatus* showed that it was predominantly composed of monoterpenes (69.62%) and sesquiterpenes (22.73%). The antibacterial assay showed that the oil had significant inhibitory effects against both Gram-negative and Gram-positive bacteria isolates. The antibacterial activity can be attributed to the combination of several components of the oil. The results indicate that the *C. pinnatus *might be exploited as natural antibacterial agent and have application in the treatment of several infectious diseases caused by these bacteria. 
